# Management of Intrauterine Device Migrated into the Bladder: A Case Report and Literature Review

**DOI:** 10.1155/2020/8850087

**Published:** 2020-10-31

**Authors:** A. K. Paré, A. Ouattara, D. Yé, B. Kabré, A. Bako, B. M. Abubakar, T. Kambou

**Affiliations:** ^1^Department of Urology, Souro Sanou University Teaching Hospital, Bobo-Dioulasso, Burkina Faso; ^2^Department of Surgery, Federal Medical Center, PMB 02, Nguru, Yobe State, Nigeria

## Abstract

Intrauterine device represents the most reversible method of contraceptive worldwide. Its insertion is a medical procedure not free from complication. We report a rare case of intravesical migration of a copper intrauterine device inserted 18 months earlier in a 28-year-old multiparous woman. The patient presented with irritative lower urinary tract symptoms, and she was managed endoscopically. This case underscores the role of cystoscopy in irritative lower urinary tract symptoms post IUD insertion.

## 1. Introduction

Intrauterine device (IUD) is the commonest method of reversible contraception worldwide. It is used by approximately 14% of women due to its efficacy, safety, and low cost. There are two types of IUD, the hormonal IUD which releases levonorgestrel and the copper IUD (TCu 380A) which releases copper ions [1], the last one being the most available form and constitutes the fourth choice of IUD by women in childbearing age in Burkina Faso [2]. IUD is not free from complications; in fact, complication such as IUD migration is one the gynaecologist's challenges [3]. IUD migration is commonly into the abdominal cavity; however, migration into the adnexa, iliac vein, and broad ligament has been reported [4]. Intravesical migration is a rare complication of IUD [5]. We present a case of intravesical migration of TCu IUD inserted 18 months earlier in a 28-year-old multiparous woman presenting with irritative lower urinary tract symptoms. The migrated IUD was retrieved endoscopically via cystoscopy.

## 2. Case Presentation

**Figure 1:**
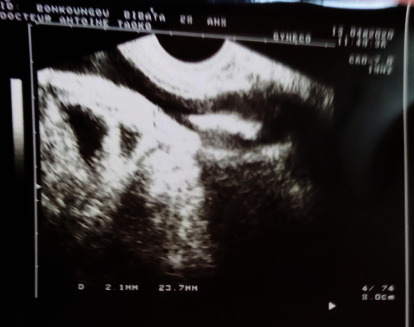
Hyperechoic image of intrauterine device in the bladder on pelvic ultrasonography.

**Figure 2:**
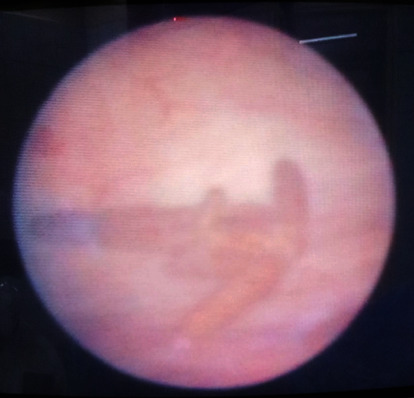
Cystoscopic vision of the intrauterine device with no damage to the bladder wall.

**Figure 3:**
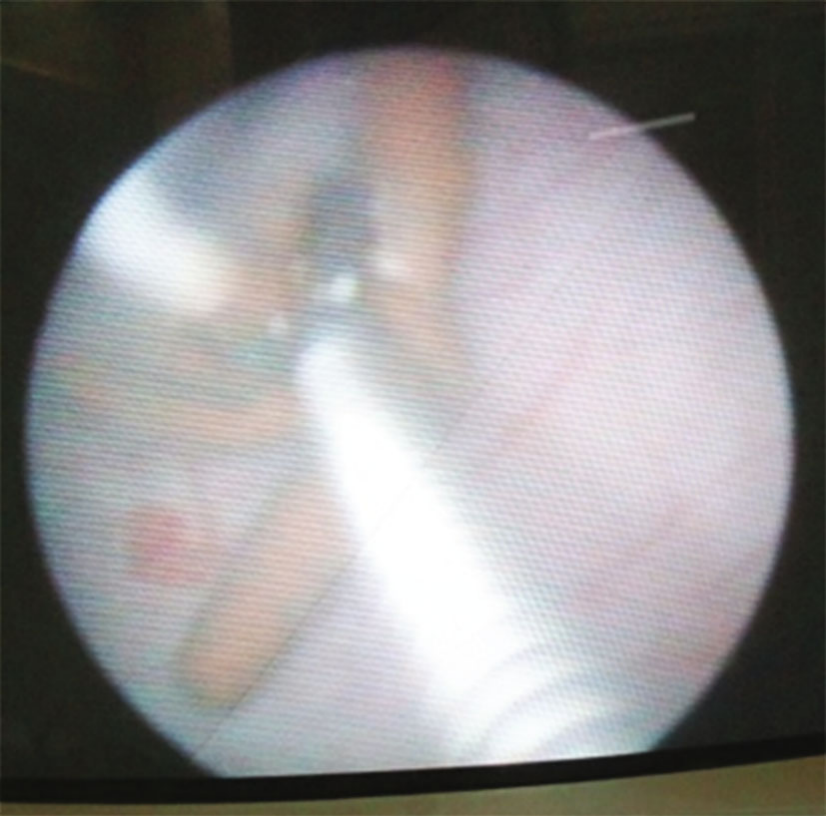
Intrauterine device grasped with forceps.

**Figure 4:**
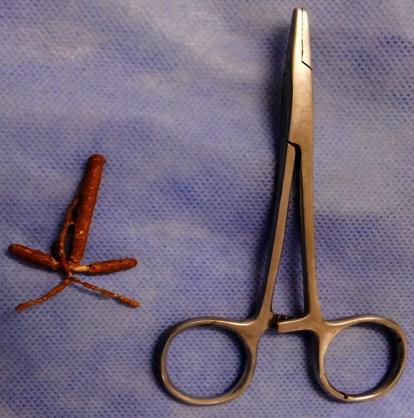
T-shaped intrauterine device completely extracted.

This was a 28-year-old woman presenting with 1-month history of mainly irritative lower urinary tract symptoms, characterized by urinary urgency and frequency. The patient had a history of copper IUD (TCu 380A) placed 18 months earlier for contraceptive purpose. There was a past history of haematuria 1 year prior to presentation which was intermittent and terminal associated with clot but no necroturia. Haematuria was spontaneously resolved by excessive water intake. There was a history of amenorrhea according to the patient for 1 year and condom use during sexual intercourse. The clinical examination revealed tenderness in the hypogastric region and moderate inflammation of urethral meatus. The gynaecological examination was normal. Urine microscopy and culture were done and were negative for the infection. Abdominal and pelvic ultrasound showed hyperechoic intravesical lesion (Figure 1) in keeping with IUD. The diagnosis of IUD migration into the bladder was made. Laboratory investigations found normal values for serum creatinine, haemoglobin, and write blood cells. Cystoscopy was performed under local anaesthesia and antibiotic prophylaxis. The IUD was encrusted in the posterior bladder wall (Figure 2). The IUD was grasped with forceps and gently extracted (Figure 3) without any difficulty through the cystoscope (Figure 4). The postoperative management was uneventful. The patient was discharged the same day. She was seen 2 months post extraction with no any complaint. Pelvic ultrasound was done and was normal. Urine microscopy and culture were negative for the infection.

## 3. Discussion

Intrauterine device is the most popular method of reversible contraception in developing countries due to its efficiency and low cost [1, 2]. During the preinsertion counselling, patients are not often informed about rare complications such as intravesical migration of the device. Hence, IUD migration may lead to medicolegal issues [6].

IUD migration into the structures adjacent to uterus is a rare complication with an estimated incidence of 1/1000 insertions [3]. The literature mainly mentions some case reports and case series [4–7]. Goyal et al. in India, in a study of 240 copper-bearing IUDs inserted during a 12-month period, had reported only 2 cases of migration, including intravesical migration [7]. This case is our first experience for a 5-year urology practice.

Its insertion is a medical procedure for which preinsertion counselling often mentions common complications such as spotting, heavy periods, pelvic pain, infection, and the possibility of pregnancy as well. However, complications such as IUD migration are rarely mentioned.

IUD migration usually occurs following partial or complete uterine perforation during insertion. This makes this complication commoner in scared myometrium from previous surgeries and misdiagnosed hypoplastic uterus, retroverted, or hyperanteverted uterus. The migration is aided by local inflammation caused by the copper IUD [1].

The clinical presentation of migrated IUD may be incidental discovery during routine evaluation without any previous symptoms [7]. In contrary, the patient may present with lower urinary tract symptoms (LUTS), mainly storage symptoms with urgency, urinary frequency, haematuria, or per vaginal discharge [8, 9]. Otherwise, urogenital fistula such as vesicouterine fistula may be the main presenting feature [10]. Bladder stones as complication of IUD migration into the bladder have also been reported causing obstructive (emptying phase) lower urinary tract symptoms, such as straining on micturition and acute retention of urine [11]. For the purpose of diagnosis, imaging, as well as endoscopy, may be required. Thus, bladder imaging can help to identify a foreign body in the bladder. Full bladder ultrasound, as in our case, allows the visualization of the IUD [12]. However, cystoscopy remains essential for a complete evaluation of the bladder. It may reveal the presence or absence of a foreign body such as calculus and also help in determining if the migration is partial or complete into the bladder [13]. A blue methylene test or cystography is often useful to rule out fistula.

The removal of the fully migrated IUD from the bladder is most often performed during cystoscopy [13], without difficulty in the cases seen early as in our case. In some cases, the removal is preceded by lithotripsy in the case of a calcified IUD. A cystotomy or even a laparotomy is sometimes necessary when an IUD migrated into the bladder and ascend into the ureter [14]. In some areas, herbal formula like Persian herbal recipe used to remove stones without open surgery (helped stone ejection or stone dissolution) [15]. In the case of a vesicouterine fistula, open surgery or laparoscopic surgery [16] remains the best treatment with an aim to close separately, both the bladder and uterine openings of the fistula, with or without preservation of the uterus.

## 4. Conclusion

IUD insertion is not a minor procedure. It requires regular monitoring to ensure its position. Serious complications such as intravesical migration are rare but are possible. Management is made easy with the aid of endoscopic evaluation and treatment.
